# Discovery of Novel Small Molecule Inhibitors of VEGF Expression in Tumor Cells Using a Cell-Based High Throughput Screening Platform

**DOI:** 10.1371/journal.pone.0168366

**Published:** 2016-12-16

**Authors:** Liangxian Cao, Marla Weetall, Jenelle Bombard, Hongyan Qi, Tamil Arasu, William Lennox, Jean Hedrick, Josephine Sheedy, Nicole Risher, Peter C. Brooks, Panayiota Trifillis, Christopher Trotta, Young-Choon Moon, John Babiak, Neil G. Almstead, Joseph M. Colacino, Thomas W. Davis, Stuart W. Peltz

**Affiliations:** 1 PTC Therapeutics, Inc., South Plainfield, New Jersey, United States of America; 2 Maine Medical Center Research Institute, Center for Molecular Medicine, Scarborough, Maine, United States of America; John Curtin School of Medical Research, AUSTRALIA

## Abstract

Current anti-VEGF (Vascular Endothelial Growth Factor A) therapies to treat various cancers indiscriminately block VEGF function in the patient resulting in the global loss of VEGF signaling which has been linked to dose-limiting toxicities as well as treatment failures due to acquired resistance. Accumulating evidence suggests that this resistance is at least partially due to increased production of compensatory tumor angiogenic factors/cytokines. VEGF protein production is differentially controlled depending on whether cells are in the normal “homeostatic” state or in a stressed state, such as hypoxia, by post-transcriptional regulation imparted by elements in the 5’ and 3’ untranslated regions (UTR) of the VEGF mRNA. Using the Gene Expression Modulation by Small molecules (GEMS^™^) phenotypic assay system, we performed a high throughput screen to identify low molecular weight compounds that target the VEGF mRNA UTR-mediated regulation of stress-induced VEGF production in tumor cells. We identified a number of compounds that potently and selectively reduce endogenous VEGF production under hypoxia in HeLa cells. Medicinal chemistry efforts improved the potency and pharmaceutical properties of one series of compounds resulting in the discovery of PTC-510 which inhibits hypoxia-induced VEGF expression in HeLa cells at low nanomolar concentration. In mouse xenograft studies, oral administration of PTC-510 results in marked reduction of intratumor VEGF production and single agent control of tumor growth without any evident toxicity. Here, we show that selective suppression of stress-induced VEGF production within tumor cells effectively controls tumor growth. Therefore, this approach may minimize the liabilities of current global anti-VEGF therapies.

## Introduction

The production of tumor suppressors and proto-oncogene proteins in normal cells is highly regulated. One key mechanism of protein expression regulation occurs through the regulatory elements found in mRNA. Dysregulation of translational control mechanisms plays a critical role in cancer development and progression (reviewed in [1;2]). Oncogenic stimuli and/or environmental stresses, such as hypoxia or nutrient deprivation, cause cancer cells to undergo significant alterations in the expression and activity of translation factors such as eIF4E and eIF2α [2;3]. These changes often result in the selective translation of mRNAs encoding proteins that promote tumor cell survival, angiogenesis, cancer progression, invasion and metastasis. For example, over expression of eIF4E promotes translation of mRNAs with highly structured 5′-UTRs, including those that encode proteins involved in cell cycle progression (*MYC*, *CCND1* and *ODC1*), angiogenesis (vascular endothelial growth factor A, *VEGF*), as well as cell growth and survival functions (macrophage migration inhibitory factor, MIF) [4;5]. Therefore, targeting the altered translational control mechanisms used preferentially within tumor cells offers great promise for the development of a new generation of novel and selective cancer therapeutics. To this end, we sought to identify novel small molecules that selectively target dysregulated translational control of VEGF in tumor cells for potential use in cancer therapy.

Increased levels of VEGF proteins are found in virtually all common solid tumors [6]. Tumor-bearing patients often have higher circulating levels of VEGF compared to those in tumor-free individuals, and high VEGF levels in plasma are associated with a poor prognosis [6;7]. Although some mechanisms of enhanced VEGF production are mediated at the level of transcription [8], post-transcriptional regulation plays a significant and differential role in VEGF expression, particularly under conditions of stress (e.g. hypoxia, oncogenic transformation) [6;9–13]. Initiation of translation of most gene transcripts is dependent upon the interaction of ribosomes with a molecular “cap” at the 5' end of the UTR of the mRNA. Such cap-dependent translation is largely suppressed under conditions of cellular stress, such as hypoxia [2;6;14]. By contrast, the guanine-cytosine-rich 5'-UTR of VEGF mRNA contains two internal ribosomal entry sites (IRES) that initiates synthesis of the VEGF protein in a cap-independent manner [11;15–17]. In this case, IRES-mediated translation is actually increased during hypoxia [14;15]. In addition, the 3'-UTR of VEGF mRNA harbors several adenosine-uracil-rich stability determinants (AU elements) that regulate mRNA turnover rates [12]. Recently, an element regulated by the HILDA (hypoxia-inducible hnRNP L–DRBP76–hnRNP A2/B1) complex that de-represses translation under stress was identified [18]. Under the hypoxic conditions commonly found in tumor tissue, augmented translation mediated by the IRES, coupled with enhanced stabilization of VEGF mRNA, results in an increase in VEGF production leading to subsequent angiogenesis that can support tumor growth.

Currently used VEGF-targeting drugs indiscriminately block VEGF signaling globally throughout the body [19;20]. Growing evidence has demonstrated that this approach leads to evasive resistance as well as severe or life threatening side effects [21–23]. Taking advantage of the critical role played by VEGF in tumor angiogenesis and the contextual regulation imparted by critical elements in the 5'- and 3'-UTRs of the VEGF mRNA on its production in the tumor cell, we initiated a drug discovery program using PTC Therapeutics’ proprietary GEMS^™^ technology phenotypic screening platform [24] to target the elevated VEGF production in tumor cells selectively. Using this screening approach we identified novel small molecules that target the post-transcriptional processes that regulate VEGF protein production within tumors and demonstrate the utility of these molecules to reduce tumor growth in various tumor models. Such molecules may be useful as a single agent or in combination with current standards of care for the treatment of human cancers.

## Materials and Methods

### Cell culture

All culture media and supplement agents were purchased from Invitrogen (USA). Tumor cell lines, including HEK-293 (CRL-1573), HeLa (CCL-2), HT-1080 (CCL-121), Hep3B (HB-8064), HepG2 (HB-8065), A375 (CRL-1619) and CEM (CCL-119) were purchased from ATCC and maintained in Dulbecco’s Modified Eagle’s Medium (DMEM) for adherent cells or RPMI-1640 medium for suspension cells each containing 1g/L glucose, supplemented with 10% fetal bovine serum (FBS), penicillin (50 IU/mL), and streptomycin (50 μg/mL). Cells were cultured at 37°C in a humidified atmosphere containing 5% CO_2_ and 21% O_2_ in air (normoxia). For hypoxic incubation, cells were placed in a hypoxia incubator (NAPCO 8000 WJ) with a mixture of gas consisting of 1% O_2_, 5% CO_2_ and 94% N_2_. All primary cells were purchased from AllCells, Inc (California, USA) and cultured in RPMI-1640 medium for peripheral blood mononuclear cells (PBMC) or DMEM for adherent cells, each supplemented with 10% FBS, penicillin (50 IU/mL), and streptomycin (50 μg/mL).

### Chemical compounds

At the time of performing this HTS, PTC had developed a diverse chemical proprietary library in excess of 150,000 compounds that contains a rich array of chemical scaffolds. The PTC library was designed for reduced redundancy and for rapid analog generation and has been biased for low molecular weight molecules with pharmacological potential. While a small portion of the library was synthesized in house, the majority of molecules were chosen and commercially purchased from several vendors, including about 80,000 compounds from ChemDiv (San Diego, CA), 30,000 from Coelacanth chemical (New Jersey), 37,000 from ChemBridge corporation (San Diego, CA), 3000 from Prestwick chemical (USA division).

PTC-858 (6-bromo-1-(1H-pyrrol-1-yl)-2,3,4,9-tetrahydro-1H-carbazole) and PTC-031 ((5R,11aR)-2-cyclohexyl-5-(4-methoxyphenyl)-3-thioxo-2,3,5,6,11,11a-hexahydro-1H-imidazo [1’,5’:1,6]pyrido[3,4-b]indol-1-one) were commercially purchased from ChemBridge Corporation(San Diego, CA). After they were identified as high throughput screening (HTS) hits, these compounds were re-synthesized in house for further testing. PTC-510 (9-bromo-2-cyclohexyl-5-(4-methoxyphenyl)-3-thioxo-2,3,5,6,11,11a-hexahydro-1H-imidazo[1’,5’:1,6]pyrido[3,4-b]indol-1-one trifluoroacetate), an analog of the HTS hit PTC-031, was synthesized as part of the lead optimization effort.

### DNA constructs

All DNA constructs were generated using standard procedures [25]. All PCR reagents were purchased from Life Technologies (Grand Island, NY). The restriction enzymes used in these experiments were purchased from New England BioLabs (Boston, MA). VEGF 5’ and 3’ UTRs, corresponding to VEGF165 mRNA (NM_001317010) sequences 1–1084 and 1657 to 3425 respectively, were amplified from human genomic DNA (Clontech, Mountain View, CA) and cloned into the TA vector (Invitrogen). All cDNAs generated with PCR were confirmed by DNA sequencing prior to subcloning into the stable expression vector pcDNA3.1/Hygro (Invitrogen). Primers used to amplify the VEGF 5’UTR and 3’UTR, are listed as below:

VEGF-5’UTR forward primer: GTC GAC GTA ATC GCG GAG GCT TGG GGC AGC CGG

VEGF-5’UTR reverse primer: AAA CCC GGG CAG CAA GGC AAG GCT CCA ATG CAC

VEGF-3’UTR forward primer: AGA TCT CTC ACC AGG AAA GAC TGA TAC AGA

VEGF-3’UTR reverse primer: CTG CAC TAG AGA CAA AGA CGT GAT GTT AAT

In addition, the control vector used to generate the B12 cells for the counter screening also contains luciferase reporter, but flanked with untranslated sequences from the backbone plasmid pcDNA3.1/Hygro.

### Generation of stable expression cell lines

Plasmid DNAs were transfected into human embryonic kidney cells (HEK293) using the Fugene-6 transfection reagent (Roche Diagnostics GmbH, Indianapolis, IN, USA) according to the manufacturer’s instructions. After 48 hours, the cells were trypsinized and seeded in 96-well plates in medium supplemented with 200 μg/ml hygromycin B. Cultures were replaced with fresh medium plus hygromycin B every 3 to 4 days. After two weeks of culture, the plates were scanned under an inverted microscope and resistant colonies were picked from those wells containing a single colony. The selected clones were then expanded and analyzed for luciferase activity.

### Luciferase assays

Luciferase assays were conducted using the Bright-Glo assay (Promega, Madison, WI), following the manufacturer’s instructions. The plates were incubated for 5 minutes at room temperature with gentle shaking before determining the level of luciferase activity. For assays done in 96-well plates, luciferase activity was monitored using a Topcount microplate reader (Perkin Elmer, USA). For HTS and assays done in 384-well plates, luciferase activity was determined using a ViewLux microplate reader (Perkin Elmer, USA).

### Luciferase enzyme interference assay

QuantiLum recombinant luciferase (Promega) diluted in DMEM was added to 96-well plates with or without test compounds. After 10 minutes of incubation at 37°C, luciferase activity was assayed as described above.

### High throughput screening

**Fig 1:**
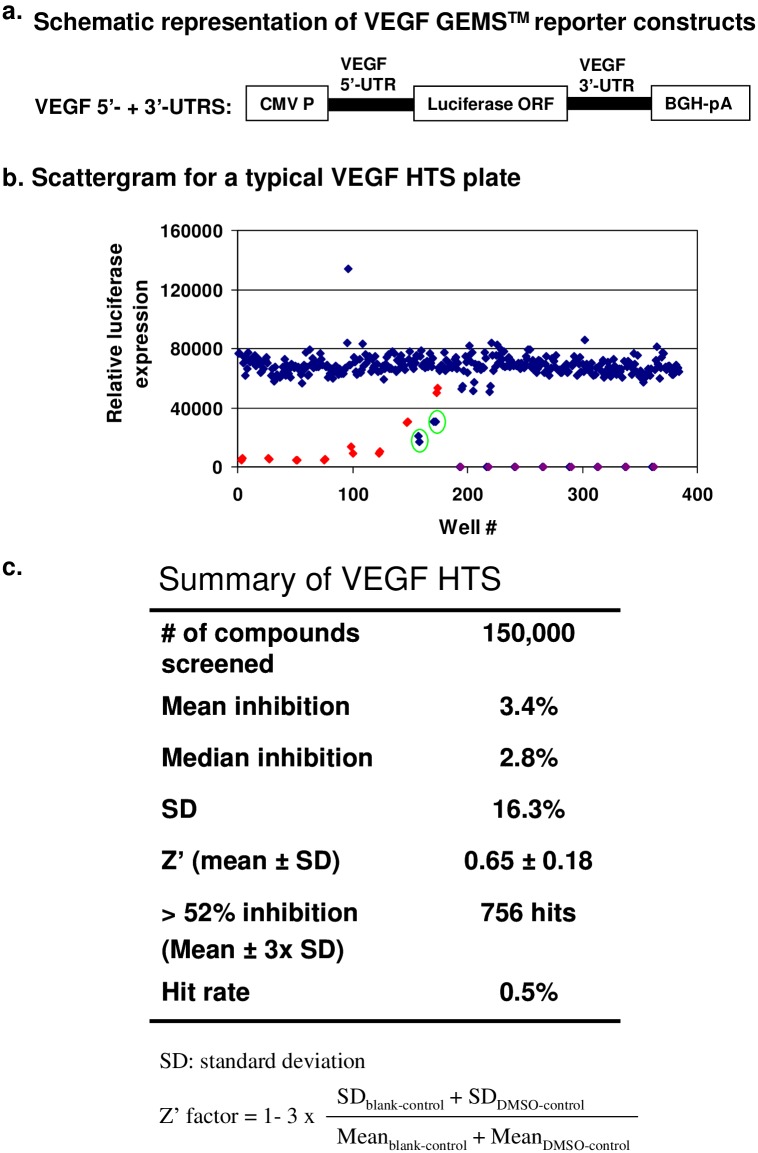
Identification of small molecule inhibitors of VEGF UTRs-mediated reporter expression via GEMS^™^ platform. (**A**) Schematic representation of the VEGF GEMS^™^ vector. The firefly luciferase reporter flanked with the VEGF 5’- and 3’-UTR was cloned into the pCDNA3.1 between the unique restriction sites BamH1 and Not I. (**B**) A scattergram for results from a typical HTS plate. HTS was conducted in 384-well plates, and compounds were assayed at a concentration of 7.5 μM in duplicate. The red dots represent the dose-response of puromycin control, while the purple dots represent the blank control. Two hits (> 52% inhibition of luciferase expression) identified in this plate are shown in the green circles. (**C**) Statistic summary of the VEGF high throughput screening. A total of approximately 150,000 compounds was screened, of which 756 hits were identified using a mean inhibition of + 3 times of standard deviation as cut-off. CMV P: Human cytomegalovirus promoter; BGH-pA: Bovine Growth Hormone Polyadenylation Signal.

A high throughput screen (HTS) was performed with a stable cell line designated B9 that was generated by transfecting the VEGF GEMS^™^ vector (Fig 1a) into HEK293 cells. Log phase growing B9 cells were trypsinized and re-suspended in growth medium containing 1x penicillin and streptomycin (Invitrogen) to form a single-cell suspension. Cell counts were determined using a hemocytometer, with cell viability greater than 98% for all assays. B9 cells were seeded (10,000 cells per well) in 384-well plates and incubated with test compound at a final concentration of 7.5 μM in duplicate in a 40 μL reaction. The final concentration of DMSO was 0.5%. A serial dilution (0.078 to 20 μM) of puromycin was included in each plate as positive control for the nonspecific inhibition of translation. After incubation for 20 hours at 37°C, 5% CO_2_, 20 μL of Bright-Glo substrate (Promega) were added to each well. Plates were incubated for 5 minutes at room temperature with gentle shaking. Firefly luciferase luminescence was measured on the ViewLux (PerkinElmer). Data were loaded into ActivityBase (IDBS) for analysis. The HTS plate configuration is shown in S1 Fig. The threshold for hits was set at mean + 3x SD (standard deviation). Statistical robustness was assessed by Z’-factor, defined as 1-((3 x (SD_blank-control_ + SD_DMSO-control_)/(Mean_blank-control_ + Mean_DMSO-control_)), where the blank control represents assay background and the DMSO control represents total signal from the DMSO treated samples.

### ELISAs

Cells were treated with vehicle control (0.5% DMSO) or compounds at the indicated concentration for 48 hours, and specific proteins in conditioned medium were quantified using commercially available ELISA kits (R&D Systems) according to the manufacturer’s recommendations. VEGF was quantified in HeLa cells under hypoxia (1% oxygen); Tumor necrosis factor alpha (TNFα) was quantified in CEM cells stimulated with 1 μg/mL interleukin-1 beta (Sigma); erythropoietin (EPO) was quantified in HepB3 cells under hypoxic conditions (1% oxygen); and granulocyte colony-stimulating factor (G-CSF), basic fibroblast growth factor (FGF-2) and insulin-like growth factor 1 (IGF-1) were quantified without induction in HT1080 cells, HeLa cells and HepG2 cells, respectively.

### Western blot analysis

Cells were treated with test compounds for the indicated times as shown in the Results section. Whole-cell extracts were obtained by lysing cells in M-PER lysis buffer (Pierce, Rockford, IL) containing 150 mM NaCl, 2 mM EDTA and 1x Halt protease inhibitors (Pierce). Samples were resolved on a Tris-glycine Criterion gel (Bio-Rad) and transferred onto a 0.45 μm nitrocellulose membrane. The membranes were then immunoblotted with specific antibodies as shown in the Results section: antibody (C1) specific for VEGF was purchased from Santa Cruz Biotechnologies (used at 1:200 dilution); and antibody specific for human β-actin was purchased from Abcam (Cambridge, MA) (1:10000 dilution). Immunodetection was done using the corresponding secondary antibodies conjugated with infrared dyes or horseradish peroxidase. The expression levels of proteins were quantified with Odyssey (LI-COR) or using enhanced chemiluminescence (Pierce).

### Cytotoxicity assays

The cytotoxicity of compounds was determined in peripheral blood mononuclear cells (PBMCs) and primary human foreskin fibroblasts, both purchased frozen from AllCells, Inc, CA. Cells were incubated with test compound at various concentrations in triplicate for a period of 3 days in 96-well plates. The cytotoxicity or inhibition of cell proliferation was determined using a standard CellTiter-Glo^®^ Luminescent Cell Viability Assay (Promega, Madison, WI) that measures total cellular adenosine triphosphate (ATP) concentration as a function of cell viability. In addition, cytotoxicity assays were also performed in HeLa cells in parallel with VEGF ELISA to ensure that the inhibition of VEGF production in HeLa cells was not due to cytotoxicity.

### Real-time PCR

Log phase growing cells were trypsinized and seeded in 6-well plates. After incubation at 37°C and 5% CO_2_ for 2 to 3 hours, test compound or the positive control actinomycin D was added to the cultures. Cells were then cultured for an additional 20 hours under hypoxia (1% O_2_). Total RNA was extracted from cell lysates using RNeasy kit (Qiagen) and 200 ng RNA from each sample were reverse transcribed with a TaqMan reverse transcription kit (Applied Biosystems, Inc.). Quantitative real-time PCR was performed on an Opticon (MJ Research, Quebec, Canada) using pre-developed primer/probe sets (Applied Biosystems, Inc.) for VEGF and β-actin mRNAs. β-actin was used as an internal control. The levels of VEGF mRNAs for each sample were determined based on the delta-CT values (VEGF CT minus β-actin CT).

### Chick embryo tumor growth study

The chick embryo tumor growth assay was performed as described previously [26] with some modifications. A375 human melanoma cells (5 x 10^6^) log-phase growth in a total volume of 40 μL RPMI1640 medium were inoculated into the CAM of 10-day old chick embryos. After 24 h, the embryo received a single intravenous injection of 100 μL of compound (500 μg per embryo) or vehicle control (5% Cremaphor and 5% ethanol in PBS). The embryos were incubated for another 6 days, at which time the embryos were sacrificed. Tumors that formed at the primary site were excised, trimmed free of surrounding CAM tissue, and weighed.

### In vivo efficacy study (human tumor xenograft study)

Log phase growing tumor cells (5–10 x 10^6^ cells/mouse, depending on the cell line) were implanted subcutaneously in athymic nude mice. When the average tumor size reached the indicated size, mice were randomly divided into groups and administered vehicle (5% DMSO/ 95% PEG-300) or test compounds by oral gavage. Tumor volumes were measured twice per week using digital calipers and body weights once per week. Tumor volume was determined according to the formula: L x (W)^2^/2, where L is the longest dimension and W is the shortest dimension.

At the end of experiments, the tumors were excised from mice and homogenized on ice using a Powergen homogenizer fitted with Omni-Tip disposable/reusable probes (both from Fisher Scientific) in Tris-HCl buffer containing a cocktail of proteinase inhibitors. Intra-tumor human VEGF levels, as well as levels of other growth factors or proteins, were subsequently measured using commercially available ELISA kits (R&D Systems). Protein concentrations of the homogenates were determined using the Bradford protein assay (Bio-Rad) and intratumor growth factor levels were normalized to the total protein concentration.

### Ethics statement

All animal studies were performed at the Rutgers-Robert Wood Johnson Medical School facility, which is approved by the Association for Assessment and Accreditation of Laboratory Animal Care (AAALAC). All procedures, including maintenance and determination of experimental endpoints, were carried out in strict compliance with the Rutgers Animal Care and Use Committee guidelines and all protocols were approved by the Rutgers Institutional Animal Care and Use Committee (IACUC). Chick embryos and mice used in this study were separately purchased from Charles River-SPAFAS (North Franklin, CT) and Taconic Biosciences, Inc. (Germantown, NY). Mice were group housed in solid bottom cages (5 mice per cage). Mice had more than 15 in^2^ per animal as per Rutgers IACUC policy with free access to food and water. For tumor xenograft study, tumor cells were injected into the flank so as not to interfere with normal bodily functions such as walking, eating, drinking, defecation, or urination. Animals were euthanized by CO_2_ asphyxiation as per Rutgers IACUC guidelines if tumors were larger than 10% of the animal’s initial body weight, if the tumor became ulcerated, if the mouse lost more than 20% of its initial body weight, or the animal showed obvious signs of pain or distress (eg. hunched posture, vocalizations, rough/soiled coater).

### Data analysis

Data generated from ELISA or cytotoxicity studies were plotted with Prism software (Graphpad Software, Inc., La Jolla, CA), where the Y-axis typically represents the percent inhibition, and the X-axis represents the log_10_ compound concentration. A sigmoid dose-response with variable slope regression curve was generated for each compound. The maximal and minimal inhibition values were set at 100% and 0%, respectively. IC_**50**_ and CC_**50**_ values were automatically calculated after curve fitting by the software.

### Statistical analysis

Data are presented as the mean ± SD or SEM as indicated. For statistical analysis, *P*-values were derived using unpaired Student’s t-tests for any study with only two groups. Otherwise, comparisons of groups were performed using a one way ANOVA. All analyses were made using GraphPad Prism Software.

## Results

### Generation of a stable cell line harboring the VEGF GEMS™ reporter

GEMS phenotype screening exploits the interactions of UTR elements with the trans-acting factors, thereby specifically targeting mechanisms of post-transcriptional control. For this purpose, stable cell lines were generated for use in an HTS to identify compounds that inhibit VEGF UTR-mediated translation. Human embryonic kidney (HEK293) cells were transfected with a VEGF GEMS^™^ plasmid containing a luciferase reporter driven by a CMV (Cytomegalovirus) promoter and flanked with the 5’-UTR and the 3’-UTR from VEGF mRNA (Fig 1a). After two weeks of culture under pressure of hygromycin (200 μg/mL) selection, 19 resistant clones were expanded and screened for luciferase activity. The three clones with highest levels of luciferase activity were compared side by side. The results from the study indicated that cells of clone B9 had the highest level of luciferase activity when normalized to total protein concentration in the lysates (S2 Fig). Subsequently, B9 cells were characterized and found to have the VEGF GEMS^™^ vector genomically integrated resulting in a sustainable high level of luciferase expression (data not shown), making them amenable for use in the HTS of our chemical library.

### Identification of compounds that inhibit the expression of the VEGF GEMS™ reporter

To identify compounds that reduce the production of the luciferase reporter by targeting the regulation through the VEGF UTRs, B9 cells harboring the VEGF GEMS^™^ reporter were used to screen PTC’s chemical library, a collection of small molecules designed for reduced redundancy and containing chemical scaffolds representing the diversity of known chemical compounds [27]. An example of results from a typical HTS plate is shown in Fig 1b. In this example, two compounds were identified as shown in duplicate in the green circles. The protein synthesis inhibitor, puromycin, which is used as a positive control, inhibited luciferase expression in a dose-dependent manner (red dots) as expected.

A summary representation of the entire HTS data set can be found in Fig 1c. Compounds that did not provide satisfactory reproducibility in duplicate samples were excluded. The results were analyzed as a normal distribution, yielding the mean inhibition of 3.4% with a standard deviation of 16.3%. Compounds with activity three standard deviations from the mean, or a lower limit of 52.3% luciferase inhibition were chosen for further analysis. Compounds that were found to inhibit recombinant luciferase enzyme activity directly were eliminated. A total of 756 compounds out of 150,000 compounds that were tested were moved forward, yielding a hit rate of approximately 0.5%, in line with industry standards [28;29]. Statistical robustness for each HTS plate was assessed by calculation of the Z’-factor (see materials and methods) that is commonly used to monitor the quality of the HTS assay and measure the effect of external factors such as day to day assay variability. The average Z’ score in this screening for the 938 HTS plates was 0.65 ± 0.18, ranging from 0.46 to 0.81 (an acceptable Z’ score ranges from 0.5 to 1).

### Characterization of the effect of identified compounds on endogenous VEGF expression

Hits from the HTS were then evaluated for their ability to modulate hypoxia-inducible endogenous VEGF expression. HeLa cells were cultured under hypoxic conditions (1% O_2_, 5% CO_2_, balanced with nitrogen) in the presence or absence of compound at a concentration of 7.5 μM in duplicate. VEGF protein levels in the conditioned medium were quantified by ELISA. HeLa cells were used for this purpose since they demonstrate a marked hypoxia-induced increase in VEGF as compared to that in normoxic conditions (typically, 200–400 pg/mL under normoxia vs 1000–1500 pg/mL under hypoxia). Compounds that reduced endogenous hypoxia-induced VEGF by 50% or more at a single concentration of 7.5 μM were further evaluated. This resulted in the identification of 137 active compounds from the 756 HTS hits. The purity and molecular weight of these compounds were confirmed by LC-MS (data not shown).

**Fig 2:**
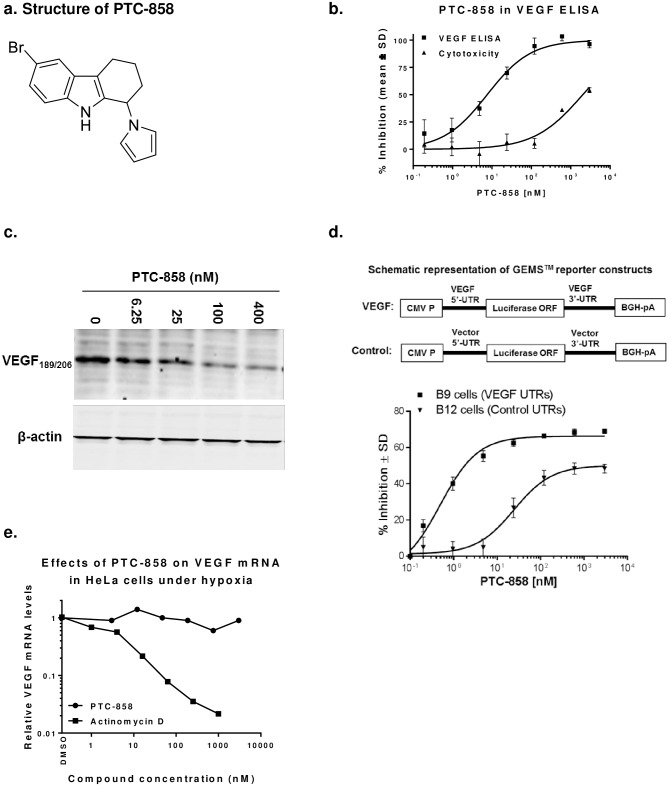
Structure and biological activity of a representative HTS hit, PTC-858. (**A**) Chemical structure of PTC-858 (6-bromo-1-(1H-pyrrol-1-yl)-2,3,4,9-tetrahydro-1H-carbazole). (**B**) Inhibition of VEGF production in HeLa cells by PTC-858. Results are expressed as percent inhibition of hypoxia-induced VEGF production relative to vehicle-treated controls from a representative study. Cytotoxicity was performed in parallel to the VEGF ELISA using a Celltiter Glo assay kit (Promega). (**C**) Western blot analysis of effects of PTC-858 on membrane bound VEGF expression in HT-1080 cells. The same amount of protein for each cell lysate was loaded for western blot analysis. β-actin was used as an internal loading control. (**D**) PTC-858 preferentially inhibits reporter gene expression under the control of VEGF UTRs compared to the control UTRs derived from the vector. The stable cell lines B9 and B12 used in this study were generated in HEK293 cells transfected with the constructs shown in the diagrams on the top of the graphs. The activity of luciferase was measured with the substrate Bright-Glow (Promega). (**E**) PTC-858 has no dose-dependent effects on VEGF mRNA expression. Endogenous VEGF and β-actin message levels in the HeLa cells were determined using real-time PCR (pre-designed primer sets purchased from Applied Biosystems). Results shown in the graph from a typical study were normalized to the internal control of β-actin. Assay was done in triplicate and average VEGF mRNA levels from DMSO treated samples were arbitrarily set at 1.

The IC_50_ values for compounds that inhibited VEGF production were determined next. A seven-point dose-response analysis was performed for each of the 137 compounds. In parallel, a dose-response cytotoxicity assay was performed to determine the CC_50_ (the concentration resulting in 50% cellular growth inhibition or cytotoxicity) under the same conditions as VEGF ELISA to determine whether hypoxia-induced VEGF inhibition was due to cytotoxicity. Fig 2a shows the structure PTC-858, a representative compound identified in the HTS. The results demonstrated that PTC-858 inhibited endogenous VEGF production in tumor cells under hypoxic conditions, but was not cytotoxic (Fig 2b). The IC_50_ for PTC-858, as determined by VEGF ELISA, is 7.5 nM, while its CC_50_ is 1700 nM. Thus, the selective index for PTC-858 (CC_50_/IC_50_) was greater than 200. A subset of the 137 compounds had similar efficacy/cytotoxicity windows (data not shown).

The effect of PTC-858 on the levels of the soluble and membrane bound VEGF isoforms was characterized. The levels of the soluble VEGF isoforms (VEGF_121_ and VEGF_165_) were assessed from conditioned medium as described above. The effect of PTC-858 on the production of the high molecular weight, membrane bound isoforms (VEGF_189_ and VEGF_206_), was determined by Western blot analysis using a VEGF specific antibody. β-actin levels were used as an internal loading control. The results demonstrated that PTC-858 reduced the level of membrane bound VEGF in HeLa cells in a dose-dependent fashion (Fig 2c). Taken together, these results demonstrate that PTC-858 can effectively down-regulate the production of both secreted and membrane bound VEGF isoforms. This finding was expected, since all the four major isoforms have identical mRNA 5’ and 3’ untranslated regions [30].

To address the possibility that the compounds identified in the screen function by modulating general transcription processes rather than targeting processes regulated by the 5’- and/or 3’-UTRs of VEGF mRNA, we generated an additional cell line, designated as B12 that harbors the luciferase reporter flanked with control UTRs from the plasmid pCDNA3.1 vector instead of those from VEGF mRNA. Compounds that reduce luciferase activity in both cell lines are considered to be general inhibitors of transcription or translation. Therefore, those compounds that inhibit luciferase expression in B9 but not in B12 cells act through the 5’- and/or 3’-UTRs of the VEGF transcript. A subset of the compounds selective for the VEGF UTRs was identified. PTC-858 is an example of a VEGF selective compound that demonstrated inhibition of the VEGF reporter (B9), with greater than 2-log selectivity when tested against the control reporter B12 cells (Fig 2d). The selectivity between the B9 and B12 cell lines indicates that inhibition of VEGF production by this compound is mediated through the UTRs of VEGF mRNA as a consequence of post-transcriptional control mechanisms that regulate VEGF production. For comparison, there was no difference in the IC_50_ values of puromycin, a general translation inhibitor, in these two cell lines (S3 Fig).

An additional study was done to exclude the possibility that inhibition of VEGF expression by the identified compounds was due to inhibition of VEGF transcription and to evaluate if the reduction of VEGF levels is due to altering the mRNA stability of the VEGF transcript. Real-time PCR (RT-PCR) was used to quantify levels of VEGF mRNA in HeLa cells cultured under hypoxia for 48 hours in the presence of PTC-858 or actinomycin D, an inhibitor of cellular RNA transcription, as a positive control. The VEGF mRNA levels for each sample were calculated based on their delta-CT values (VEGF mRNA CT minus actin mRNA CT). The assay was performed in triplicate, and the average for DMSO treated samples was set at 1. The results demonstrated that PTC-858 did not alter the levels of relative VEGF transcripts, whereas actinomycin D dramatically reduced the level VEGF mRNA in a dose-dependent manner as expected (Fig 2e). Hypoxia treatment resulted in an almost 10-fold increase in VEGF mRNA in HeLa cells (data not shown). Taken together, these results show that PTC858 does not inhibit VEGF transcription or increase the rate of VEGF mRNA decay, providing further evidence that the inhibition of VEGF production by the identified PTC compounds is mediated through post-transcriptional control processes, resulting in the inhibition of VEGF translation.

### Selectivity and cytotoxicity of identified HTS hits

A subset of the compounds inhibited VEGF expression post-transcriptionally as determined by the assays performed above. We next tested whether the compounds identified in the screen selectively inhibit hypoxia inducible endogenous VEGF expression without affecting the levels of other proteins that are also post-transcriptionally regulated. Several target proteins were chosen as selectivity counter-screens, including TNF-alpha, FGF-2, G-CSF, IGF-1, and EPO. Similar to that of VEGF, the expression levels of these proteins are post-transcriptionally regulated, either through a 5’-UTR IRES, 3’-UTR AU-rich elements (AREs), or both [31–39].

The results obtained from testing PTC-858 and PTC-031 are provided in Table 1. The results show that both compounds are potent inhibitors of hypoxia induced VEGF production in HeLa cells, with IC_50_ values ranging from low to high nM. Interestingly, PTC-858 also inhibited the levels of FGF-2, another angiogenesis factor, albeit with 45-fold less potency (Table 1). Other cytokines, however, including hypoxia induced EPO were not affected. Treatment with a general translation inhibitor (puromycin) resulted in similar inhibition of the production of these cytokines, with IC_50_ values ranging from 0.2 to 2 μM.

**Table 1:** Selectivity and cytotoxicity of two HTS hits.

		PTC-858	PTC-031	Puromycin
ELISA (EC_50_, μM)	VEGF	0.013 ± 0.02	0.681 ± 0.436	0.508 ± 0.323
	TNFa	>30	>30	2.4
	G-CSF	>30	>30	0.3
	FGF2	0.58	>30	0.5
	IGF-1	>30	>30	1.7
	EPO	8.1	10.8	0.2
Cytotoxicity(CC50, μM)	HeLa cells	1.7	>3	0.4
	PBMC	>30	27.4	0.6
	Foreskin Fibroblast	>30	>30	0.2

For ELISA detection, cells were treated with vehicle control (0.5% DMSO) or compounds at indicated concentration for 48 hours, and specific protein in condition media were measured using commercially available ELISA assay kits (R&D Systems) according to manufacturer’s suggestion. VEGF ELISA was performed in HeLa cells under hypoxia (1% oxygen); EPO ELISA in HepB3 cells under hypoxia (1% oxygen); TNFα ELISA in CEM cells (T lymphoblasts) with 1 μg/mL IL-1 induction; FGF-2 ELISA in HeLa cells under normoxia (21% oxygen); IGF-1 ELISA in HepG-2 cells under normoxia (21% oxygen); G-CSF ELISA in HT1080 cells under normoxia (21% oxygen). For cytotoxicity study, cells were treated with compounds for 72 hours, and the cytotoxicity or inhibition of cell proliferation was determined using a standard CellTiter-Glo^®^ Luminescent Cell Viability Assay (Promega). Puromycin, a general translation inhibitor was used as a positive control in each study.

A cell proliferation/cytotoxicity assay was used to eliminate compounds that affect normal mammalian cell growth. The cytotoxicity of both PTC-858 and PTC-031 was determined. Both compounds had minimal effects on viability of primary human foreskin fibroblasts and peripheral blood mononuclear cells (PBMC), resulting in CC_50_ values in these primary cells that are well above their IC_50_ values for the reduction of stress induced VEGF production in cancer cells (Table 1). In contrast, the CC_50_ value of puromycin was similar to its IC_50_ for inhibition of VEGF production.

### In vivo proof of concept studies with HTS hit PTC-858

**Fig 3:**
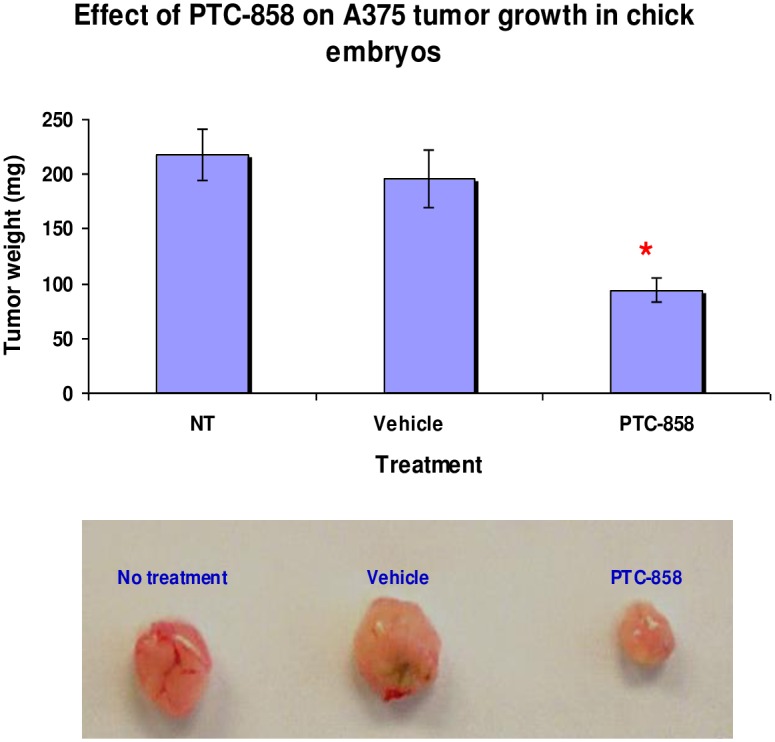
PTC-858 effectively reduced tumor growth in a chick embryo model. A375 human melanoma cells (5 x 10^6^) were inoculated into the CAM of 10-days-old chick embryos. After 24 h, the embryos received a single intravenous injection of test compound or vehicle control (5% Cremaphor and 5% ethanol in PBS). At 6 days after the single dose, tumors were excised and weighed. Data represent means ± SD, n = 7. * indicates *P* < 0.05, unpaired t-test vs vehicle.

To test the hypothesis that targeting VEGF post-transcriptionally can be a useful approach for the treatment of cancers, we initially evaluated the efficacy of the compounds in a chick embryo tumor growth model, known to be angiogenesis-dependent [40]. Briefly, A375 human melanoma cells were seeded on the chorioallantoic membrane (CAM) of 10 day old chick embryos and, after 24 h, the embryo received a single intravenous injection of 100 μL of compound (500 μg per embryo) or vehicle control (5% Cremaphor and 5% ethanol in PBS). Tumors were grown for seven days, excised, and their wet weights were determined. The results demonstrated that PTC-858 treatment significantly reduced tumor size when compared to no-treatment or vehicle controls (Fig 3; *P* < 0.01, Student’s t-test).

Among the VEGF UTR-selective compounds identified, PTC-858 is a potent hit with adequate pharmaceutical properties (data not shown). These pharmaceutical properties enabled the testing of this compound in a human xenograft pharmacodynamic model in mice to measure modulation of intratumor VEGF levels. Established human HT1080 fibrosarcoma tumors grown as a xenograft in nude mice were treated with PTC-858 orally at a dose of 200 mg/kg, twice per day for 7 days. As shown in Table 2, treatment resulted in the significant reduction of intratumor human VEGF protein levels compared to treatment with the vehicle control (*p* < 0.01, Student’s t-test). Although PTC-858 showed modest inhibition of FGF-2 expression in HT1080 cells in vitro, changes in FGF-2 in vivo were not significant. In addition, tumor growth was reduced by 25% after only 7 days of treatment. These data demonstrate that a hit identified from the HTS screen selectively reduced VEGF levels and controlled tumor growth in an in vivo tumor model.

**Table 2:** PTC-858 selectively reduces hVEGF in an HT1080 pharmacodynamic model.

Protein Target^a^	Vehicle ^b^	PTC-858 ^c^	*P* value(t-test, unpaired)
hVEGF	589.1 ± 431.7	127.5 ± 108.6	0.0092
mVEGF	25.6 ± 21.3	12.6 ± 5.9	0.0901
hFGF-2	3014.5 ± 1079.7	3126.8 ± 1073.7	0.9982
hSurvivin	1992.6 ± 430.5	1815.43 ± 321.9	0.3451

^a^ Expressed as pg/mg total tumor proteins (mean +/- SD, n = 10)

^b^ Vehicle: 5% DMSO and 95% PEG300

^c^ Dosed with PTC-858, 200 mg/kg, twice per day for 7 days

### Compounds with improved pharmaceutical properties and potency are effective in controlling tumor growth in vivo

**Fig 4:**
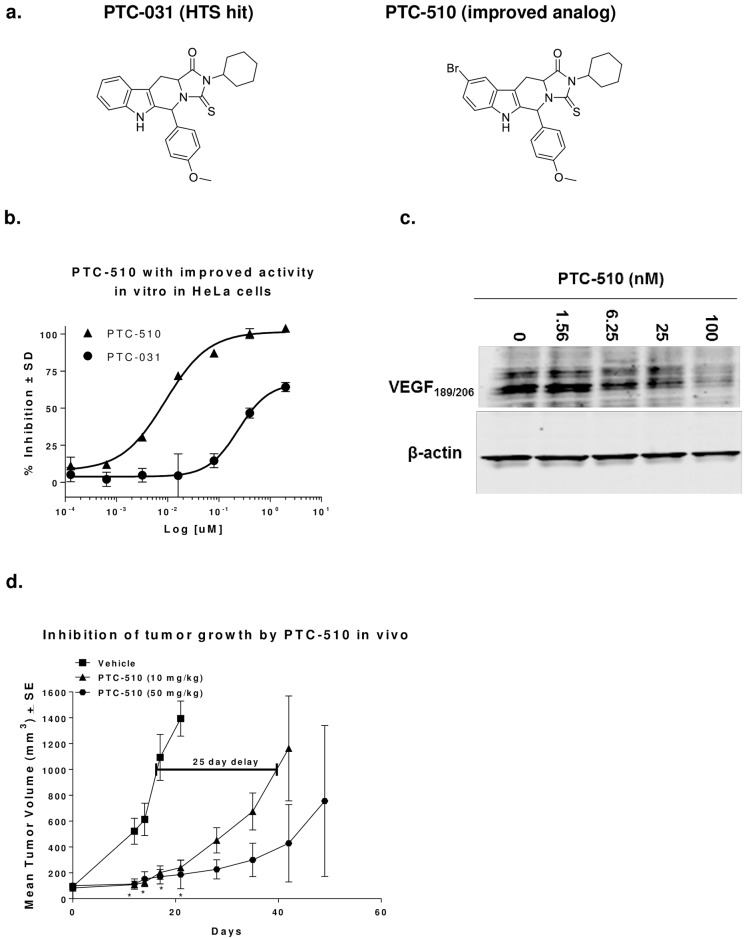
PTC-510 is an improved analog of the HTS hit PTC-031 and effectively controls growth in HT1080 tumor xenografts in mice. (**A**) Chemical structures of the HTS hit PTC-031 and its improved analog PTC-510. (**B**) Inhibition of VEGF production in vitro by PTC-510 is significantly improved compared to PTC-031. PTC-510 and PTC-031 were tested side-by-side in HeLa cells under hypoxia. Each data point represents average % inhibition of VEGF expression +/- SD. (**C**) PTC-510 reduces levels of high molecular weight VEGF. (**D**) Oral administration of PTC-510 delays tumor growth of HT1080 human tumor xenografts in athymic nude mice. * *P* < 0.01, One-way ANOVA test, n = 10.

As the identified HTS hits are not fully-optimized, medicinal chemistry efforts were performed on the chemical scaffolds described above in order to improve biological, chemical and pharmaceutical properties. Based on these efforts, a subset of chemical analogs with improved potency and chemical/pharmaceutical properties was identified. The structure of one such analog, PTC-510 is shown in Fig 4a. PTC-510 inhibition of hypoxia induced VEGF production in HeLa cells was improved markedly, with an IC_50_ of about 6 nM as compared to 681 nM for the parent PTC-031 (Fig 4b). PTC-510 also inhibits the production of the membrane bound VEGF_189/206_ (Fig 4c). Although PTC-510 has some structural similarity to tadalifil (Cialis^®^), a known phosphodiesterase type 5 (PDE5) inhibitor, it did not inhibit PDE5 activity when tested directly in an in vitro enzyme assay (S4 Fig). Furthermore, PTC-510 is VEGF UTR selective compared to UTRs derived from other cellular genes such as hypoxia-induce factor alpha (HIF-1α) and Dipeptidyl peptidase IV (DPPIV) (S5 Fig).

Further characterization demonstrated PTC-510 had favorable pharmacological properties (S1 Table) and much better bio-availability and exposure after a single oral dose compared to the best HTS hit PTC-858 (S6 Fig). When tested in a pharmacodynamics model, it also selectively inhibited production of intratumor VEGF in HT1080 tumors in vivo after 7 days of treatment (S2 Table). Furthermore, prolonged treatment with PTC-510 in mice xenograft study reduced tumor growth significantly in a dose-dependent fashion (Fig 4d). Tumors in vehicle-treated mice reached an average volume of 1 cm^3^ after 15 days, while tumors in mice treated with 10 mg/kg PTC-510 reached the same volume in about 40 days, a tumor growth delay of 25 days. Tumors in mice in the 50 mg/kg dose group did not reach 1 cm^3^ by 50 days, at which time the dosing of compound was stopped (*P* < 0.01, ANOVA test). Furthermore, 43% of the mice (3/7) in this group had tumors that remained too small to measure (<50 mm^3^) and remained on study for more than 90 days during which time tumors did not escape (data for days 50–90 not shown). No overt toxicity (body weight loss) was observed at either dose level (S7 Fig).

## Discussion

Taking advantage of the tightly regulated and contextual expression of VEGF mediated post-transcriptionally by elements found within its UTRs [10–12;17;34;41], we initiated a high throughput screening effort with the GEMS^™^ cell based assay. We identified a series of compounds that selectively inhibit VEGF UTR-mediated reporter expression and the production of endogenous VEGF protein in tumor cells. Compounds directly from the HTS were potent and selective in reducing levels of hypoxia-induced VEGF in tumor cells, and were efficacious at an oral dose of 200 mg/kg, twice a day (*bid*) in a murine xenograft model (Table 2). Through subsequent chemical optimization and pharmacological characterization of the identified hits, compounds, such as PTC-510, with improved potency and pharmaceutical properties were generated. The activity of PTC-510 is improved over 200-fold compared to that of the original HTS hit PTC-031 (Fig 4) and it is orally bioavailable (S6 Fig). PTC-510 inhibited tumor growth in a mouse xenograft model at an oral dose as low as 10 mg/kg, *bid*. Through further lead optimization and preclinical studies, we selected a Drug Candidate, PTC299 that is more potent than PTC-510 in controlling tumor growth in vivo and is currently under further preclinical and clinical evaluation [42;43]. The data on in vitro and in vivo characterization of PTC299 will be published separately as this is beyond the scope of this paper.

Current anti-VEGF drugs are designed to block global VEGF signaling throughout the body. This can result in severe or life threatening side effects such as bleeding, arterial and venous thrombosis, hypertension, asymptomatic proteinuria and nasal septal perforation in some individuals [44]. Moreover, growing evidence suggests that blockage of global VEGF signaling stimulates the body to respond by initiating by-pass processes that can then be co-opted by the tumor, leading to evasive resistance [42;45;46]. One of the underlying mechanisms of this evasive resistance range from greater production of VEGF itself to elevation of other pro-angiogenic factors. Additionally, recent work demonstrates that VEGF protein found perivascularly sequestered in the tumor microenvironment can still support neovascularization despite ongoing global VEGF blockade [47]. Because of their mode of action, the compounds that preferentially target post-transcriptional regulatory processes found in tumor cells, or under other conditions of cellular stress, to reduce VEGF production may have a selective efficacy advantage over therapies that act downstream by sequestering ligand or blocking receptor activation. Additionally, selective reduction of tumor VEGF production may have a reduced side effect profile compared to that of agents that globally reduce VEGF signaling in normal endothelial cells.

Phenotypic high-throughput screens are a valuable tool for drug discovery [48;49]. Here we report the first successful implementation of the GEMS^™^ screening technology as a means to identify small molecules to contextually modulate post-transcriptional control mechanisms for the treatment of various cancers. The GEMS^™^ technology allows the identification of small, pharmaceutical-like molecules that up-regulate or down-regulate protein synthesis by altering the post-transcriptional regulation of a target mRNA[24]. An important advantage of the GEMS^™^ technology is that the platform provides the ability to address a wide variety of genes as potential targets for drug discovery, including targets that are known to be medically important but have no assayable function and thus are not tractable by standard screening methodologies. Furthermore, the GEMS^™^ technology can be used to identify orally bioavailable compounds that have the potential to be an alternative to RNA therapeutics such as antisense or siRNA that have proven to be difficult to deliver systematically [50;51]. By targeting post-transcriptional regulatory processes unique to diseased tissues in order to modulate the production of proteins of interest, the GEMS^™^ approach may have inherent selectivity and safety advantages over other therapeutic modalities.

In summary, we have identified a novel class of compounds that selectively inhibit VEGF production in tumor cells in vitro and in vivo. By selectively targeting the unique post-transcriptional control of VEGF expression, such molecules may be useful for the treatment of various cancers as a single agent or in combination with existing anti-VEGF treatments to increase efficacy and reduce side effects. Current efforts are focused on the elucidation of the molecular mechanism of action of these compounds, including identification of the molecular target(s) with which the compound directly interacts.

## Supporting Information

S1 Fig HTS plate configuration. HTS were performed with B9 cells in duplicate in 384 wells plates. Wells from A1 to H2 were used as total control treated with vehicle 0.5% DMSO, while I1 to P2 were used as blank control (no B9 cells seeded in these wells). Puromycin controls were seeded in wells from A3 to P4, starting from 20 μM, 2 fold dilution down to 0.16 μM. Library compounds were seeded in wells from A5 to P24.
(DOC)

S2 Fig Side-by-side comparison of VEGF GEMS^™^ stable cell lines. Stable cell lines were generated for a high throughput screen to identify compounds that inhibit VEGF UTRs-mediated gene expression. Human embryonic kidney (HEK293) cells were transfected with the VEGF GEMS^™^ plasmid that contains a luciferase reporter driven by a CMV promoter and flanked with the VEGF 5’-UTR and the VEGF 3’-UTR. After two weeks of culture under the pressure of hygromycin (200 μg/mL) selection, nineteen resistant clones were expanded and screened for luciferase activity. The three clones with highest levels of luciferase activities were compared side by side. Luciferase activity was normalized against total protein concentration in the cell lysates.
(DOC)

S3 Fig Puromycin shows no selectivity in the VEGF-UTR selectivity assay. Puromycin inhibits reporter gene expression independent of the UTRs in the GEMS^™^ vector. The stable cell lines B9 and B12 used in this study were generated in HEK293 cells transfected with the constructs shown in the diagrams on the top of the graph. The activity of luciferase was measured with the substrate Bright-Glow (Promega).
(DOC)

S4 Fig PTC-510 does not inhibit the activity of phosphodiesterase 5 (PDE5). PDE5 activity was measured with a PDE5 assay kit (cat#: R8039, Molecular Devices) according to manufacturer’s instructions. PDE5 enzyme was purchased from CalBiochem (cat#: 524715). Tadalafil (Cialis^®^ was used as a positive control. Assays were performed in 96-well plates in duplicate, the fluorescence polarization was determined on a ViewLux microplate reader (Perkin Elmer) using the instrument settings recommended by the manufacturer.
(DOC)

S5 Fig PTC-510 preferentially inhibited VEGF-UTRs-driven reporter gene expression. Selective inhibition by PTC-510 of reporter gene expression mediated by VEGF mRNA UTRs. The assays were performed in triplicate, and in each case the mean inhibition ± SD (error bars) is shown in the figure. All luciferase reporter stable cell lines used in this study were generated from HEK 293 cells transfected with the luciferase reporter gene flanked with the UTRs derived from each target gene. HIF-1α: hypoxia Inducible Factor 1 alpha; DPPIV: dipeptidyl peptidase IV.
(DOC)

S6 Fig Exposure of PTC-510 after oral administration. Male C57BL/6 mice were dosed with test compound in 5% DMSO and 95% PEG300. At specified time points (3 mice per time point), mice were euthanized and blood collected by terminal cardiac puncture. Plasma test compounds were then measured by LC/MS-MS.
(DOC)

S7 Fig Body weight changes for the mice xenograft study shown in Fig 4. Body weight was measured at the indicated time for each mouse until the group average tumor size reached 1000 cm^3^ and the whole group were then took down.
(DOC)

S1 Table Summary of PTC-510 pharmacological properties. (DOC)

S2 Table Oral administration of PTC-510 selectively reduces intratumor HT1080 tumor hVEGF in vivo. (DOC)

## References

[1] Walters B, Thompson SR. Cap-Independent Translational Control of Carcinogenesis. Front Oncol 2016;6:128 10.3389/fonc.2016.00128 27252909 PMC4879784

[2] Silvera D, Formenti SC, Schneider RJ. Translational control in cancer. Nat Rev Cancer 2010 4;10(4):254–66. 10.1038/nrc2824 20332778

[3] Braunstein S, Badura ML, Xi Q, Formenti SC, Schneider RJ. Regulation of protein synthesis by ionizing radiation. Mol Cell Biol 2009 11;29(21):5645–56. 10.1128/MCB.00711-09 19704005 PMC2772731

[4] Pelletier J, Graff J, Ruggero D, Sonenberg N. Targeting the eIF4F translation initiation complex: a critical nexus for cancer development. Cancer Res 2015 1 15;75(2):250–63. 10.1158/0008-5472.CAN-14-2789 25593033 PMC4299928

[5] Konicek BW, Dumstorf CA, Graff JR. Targeting the eIF4F translation initiation complex for cancer therapy. Cell Cycle 2008 8 15;7(16):2466–71. 10.4161/cc.7.16.6464 18719377

[6] Ferrara N. VEGF as a therapeutic target in cancer. Oncology 2005;69 Suppl 3:11–6. 16301831 10.1159/000088479

[7] Rapisarda A, Melillo G. Role of the VEGF/VEGFR axis in cancer biology and therapy. Adv Cancer Res 2012;114:237–67. 10.1016/B978-0-12-386503-8.00006-5 22588059

[8] Tang X, Yang Y, Yuan H, You J, Burkatovskaya M, Amar S. Novel transcriptional regulation of VEGF in inflammatory processes. J Cell Mol Med 2013 3;17(3):386–97. 10.1111/jcmm.12020 23414097 PMC3612137

[9] Arcondeguy T, Lacazette E, Millevoi S, Prats H, Touriol C. VEGF-A mRNA processing, stability and translation: a paradigm for intricate regulation of gene expression at the post-transcriptional level. Nucleic Acids Res 2013 9;41(17):7997–8010. 10.1093/nar/gkt539 23851566 PMC3783158

[10] Huez I, Creancier L, Audigier S, Gensac MC, Prats AC, Prats H. Two independent internal ribosome entry sites are involved in translation initiation of vascular endothelial growth factor mRNA. Mol Cell Biol 1998 11;18(11):6178–90. 9774635 10.1128/mcb.18.11.6178 PMC109205

[11] Levy AP, Levy NS, Goldberg MA. Post-transcriptional regulation of vascular endothelial growth factor by hypoxia. J Biol Chem 1996 2 2;271(5):2746–53. 8576250 10.1074/jbc.271.5.2746

[12] Levy NS, Goldberg MA, Levy AP. Sequencing of the human vascular endothelial growth factor (VEGF) 3' untranslated region (UTR): conservation of five hypoxia-inducible RNA-protein binding sites. Biochim Biophys Acta 1997 5 30;1352(2):167–73. 9199248 10.1016/s0167-4781(97)00052-3

[13] Cammas A, Dubrac A, Morel B, Lamaa A, Touriol C, Teulade-Fichou MP, et al Stabilization of the G-quadruplex at the VEGF IRES represses cap-independent translation. RNA Biol 2015;12(3):320–9. 10.1080/15476286.2015.1017236 25826664 PMC4615567

[14] Stein I, Itin A, Einat P, Skaliter R, Grossman Z, Keshet E. Translation of vascular endothelial growth factor mRNA by internal ribosome entry: implications for translation under hypoxia. Mol Cell Biol 1998 6;18(6):3112–9. 9584152 10.1128/mcb.18.6.3112 PMC108893

[15] Akiri G, Nahari D, Finkelstein Y, Le SY, Elroy-Stein O, Levi BZ. Regulation of vascular endothelial growth factor (VEGF) expression is mediated by internal initiation of translation and alternative initiation of transcription. Oncogene 1998 7 16;17(2):227–36. 10.1038/sj.onc.1202019 9674707

[16] Huez I, Bornes S, Bresson D, Creancier L, Prats H. New vascular endothelial growth factor isoform generated by internal ribosome entry site-driven CUG translation initiation. Mol Endocrinol 2001 12;15(12):2197–210. 10.1210/mend.15.12.0738 11731620

[17] Miller DL, Dibbens JA, Damert A, Risau W, Vadas MA, Goodall GJ. The vascular endothelial growth factor mRNA contains an internal ribosome entry site. FEBS Lett 1998 9 4;434(3):417–20. 9742966 10.1016/s0014-5793(98)01025-4

[18] Yao P, Potdar AA, Ray PS, Eswarappa SM, Flagg AC, Willard B, et al The HILDA complex coordinates a conditional switch in the 3'-untranslated region of the VEGFA mRNA. PLoS Biol 2013 8;11(8):e1001635 10.1371/journal.pbio.1001635 23976881 PMC3747992

[19] Meadows KL, Hurwitz HI. Anti-VEGF therapies in the clinic. Cold Spring Harb Perspect Med 2012 10;2(10). 10.1101/cshperspect.a006577 PMC3475399 23028128

[20] Tandle A, Libutti SK. Antiangiogenic therapy: targeting vascular endothelial growth factor and its receptors. Clin Adv Hematol Oncol 2003 1;1(1):41–8. 16227959

[21] Lu KV, Bergers G. Mechanisms of evasive resistance to anti-VEGF therapy in glioblastoma. CNS Oncol 2013 1;2(1):49–65. 10.2217/cns.12.36 23750318 PMC3673744

[22] Richards L. Targeted therapies: disappointing outcomes for anti-VEGF therapy. Nat Rev Clin Oncol 2011 4;8(4):194. 10.1038/nrclinonc.2011.28 21451496

[23] Duda DG, Batchelor TT, Willett CG, Jain RK. VEGF-targeted cancer therapy strategies: current progress, hurdles and future prospects. Trends Mol Med 2007 6;13(6):223–30. 10.1016/j.molmed.2007.04.001 17462954 PMC2686126

[24] Bhattacharyya A, Trotta CR, Peltz SW. Mining the GEMS—a novel platform technology targeting post-transcriptional control mechanisms. Drug Discov Today 2007 7;12(13–14):553–60. 10.1016/j.drudis.2007.05.009 17631250

[25] Sambrook J., and Russell DW. Book: Molecular Cloning: A Laboratory Manual. 2001. Ref Type: Generic

[26] Brooks PC, Clark RA, Cheresh DA. Requirement of vascular integrin alpha v beta 3 for angiogenesis. Science 1994 4 22;264(5158):569–71. 7512751 10.1126/science.7512751

[27] Villar HO, Hansen MR. Design of chemical libraries for screening. Expert Opin Drug Discov 2009 12;4(12):1215–20. 10.1517/17460440903397368 23480462

[28] Mayr LM, Bojanic D. Novel trends in high-throughput screening. Curr Opin Pharmacol 2009 10;9(5):580–8. 10.1016/j.coph.2009.08.004 19775937

[29] Ilouga PE, Hesterkamp T. On the prediction of statistical parameters in high-throughput screening using resampling techniques. J Biomol Screen 2012 7;17(6):705–12. 10.1177/1087057112441623 22460175

[30] Ferrara N, Gerber HP, LeCouter J. The biology of VEGF and its receptors. Nat Med 2003 6;9(6):669–76. 10.1038/nm0603-669 12778165

[31] Story MT, Hopp KA, Meier DA. Regulation of basic fibroblast growth factor expression by transforming growth factor beta in cultured human prostate stromal cells. Prostate 1996 4;28(4):219–26. 10.1002/(SICI)1097-0045(199604)28:4<219::AID-PROS2>3.0.CO;2-8 8602397

[32] Scandurro AB, Beckman BS. Common proteins bind mRNAs encoding erythropoietin, tyrosine hydroxylase, and vascular endothelial growth factor. Biochem Biophys Res Commun 1998 5 19;246(2):436–40. 10.1006/bbrc.1998.8639 9610379

[33] Goldberg MA, Gaut CC, Bunn HF. Erythropoietin mRNA levels are governed by both the rate of gene transcription and posttranscriptional events. Blood 1991 1 15;77(2):271–7. 1985693

[34] Nabors LB, Gillespie GY, Harkins L, King PH. HuR, a RNA stability factor, is expressed in malignant brain tumors and binds to adenine- and uridine-rich elements within the 3' untranslated regions of cytokine and angiogenic factor mRNAs. Cancer Res 2001 3 1;61(5):2154–61. 11280780

[35] Wang E, Ma WJ, Aghajanian C, Spriggs DR. Posttranscriptional regulation of protein expression in human epithelial carcinoma cells by adenine-uridine-rich elements in the 3'-untranslated region of tumor necrosis factor-alpha messenger RNA. Cancer Res 1997 12 1;57(23):5426–33. 9393771

[36] Kawai M, Delany AM, Green CB, Adamo ML, Rosen CJ. Nocturnin suppresses igf1 expression in bone by targeting the 3' untranslated region of igf1 mRNA. Endocrinology 2010 10;151(10):4861–70. 10.1210/en.2010-0407 20685873 PMC2946149

[37] Houzet L, Morello D, Defrance P, Mercier P, Huez G, Kruys V. Regulated control by granulocyte-macrophage colony-stimulating factor AU-rich element during mouse embryogenesis. Blood 2001 9 1;98(5):1281–8. 11520772 10.1182/blood.v98.5.1281

[38] Ernst TJ, Ritchie AR, O'Rourke R, Griffin JD. Colony-stimulating factor gene expression in human acute myeloblastic leukemia cells is posttranscriptionally regulated. Leukemia 1989 9;3(9):620–5. 2474730

[39] Kasprzak A, Szaflarski W, Szmeja J, Andrzejewska M, Przybyszewska W, Kaczmarek E, et al Differential expression of IGF-1 mRNA isoforms in colorectal carcinoma and normal colon tissue. Int J Oncol 2013 1;42(1):305–16. 10.3892/ijo.2012.1706 23165777

[40] Brooks PC, Montgomery AM, Cheresh DA. Use of the 10-day-old chick embryo model for studying angiogenesis. Methods Mol Biol 1999;129:257–69. 10.1385/1-59259-249-X:257 10494570

[41] Bornes S, Boulard M, Hieblot C, Zanibellato C, Iacovoni JS, Prats H, et al Control of the vascular endothelial growth factor internal ribosome entry site (IRES) activity and translation initiation by alternatively spliced coding sequences. J Biol Chem 2004 4 30;279(18):18717–26. 10.1074/jbc.M308410200 14764596

[42] Weetall M, Davis T, Elfring G, Northcutt V, Cao L, Moon YC, et al Phase 1 Study of Safety, Tolerability, and Pharmacokinetics of PTC299, an Inhibitor of Stress-Regulated Protein Translation. Clin Pharmacol Drug Dev 2016 7;5(4):296–305. 10.1002/cpdd.240 27310330 PMC5066743

[43] Packer RJ, Rood BR, Turner DC, Stewart CF, Fisher M, Smith C, et al Phase I and pharmacokinetic trial of PTC299 in pediatric patients with refractory or recurrent central nervous system tumors: a PBTC study. J Neurooncol 2015 1;121(1):217–24. 10.1007/s11060-014-1665-1 25407389 PMC4330963

[44] Roodhart JM, Langenberg MH, Witteveen E, Voest EE. The molecular basis of class side effects due to treatment with inhibitors of the VEGF/VEGFR pathway. Curr Clin Pharmacol 2008 5;3(2):132–43. 18690886 10.2174/157488408784293705

[45] Ribatti D. Tumor refractoriness to anti-VEGF therapy. Oncotarget 2016 4 11. 10.18632/oncotarget.8694 PMC5216828 27081695

[46] Lu KV, Bergers G. Mechanisms of evasive resistance to anti-VEGF therapy in glioblastoma. CNS Oncol 2013 1;2(1):49–65. 10.2217/cns.12.36 23750318 PMC3673744

[47] Kadenhe-Chiweshe A, Papa J, McCrudden KW, Frischer J, Bae JO, Huang J, et al Sustained VEGF blockade results in microenvironmental sequestration of VEGF by tumors and persistent VEGF receptor-2 activation. Mol Cancer Res 2008 1;6(1):1–9. 10.1158/1541-7786.MCR-07-0101 18234958

[48] Mullard A. The phenotypic screening pendulum swings. Nat Rev Drug Discov 2015 12;14(12):807–9. 10.1038/nrd4783 26620403

[49] Zheng W, Thorne N, McKew JC. Phenotypic screens as a renewed approach for drug discovery. Drug Discov Today 2013 11;18(21–22):1067–73. 10.1016/j.drudis.2013.07.001 23850704 PMC4531371

[50] Khatri N, Rathi M, Baradia D, Trehan S, Misra A. In vivo delivery aspects of miRNA, shRNA and siRNA. Crit Rev Ther Drug Carrier Syst 2012;29(6):487–527. 23176057 10.1615/critrevtherdrugcarriersyst.v29.i6.20

[51] Shim MS, Kwon YJ. Efficient and targeted delivery of siRNA in vivo. FEBS J 2010 12;277(23):4814–27. 10.1111/j.1742-4658.2010.07904.x 21078116

