# Supplementary material for: Discovery of Novel Small Molecule Inhibitors of VEGF Expression in Tumor Cells Using a Cell-Based High Throughput Screening Platform
Source: PLoS One. 2016 Dec 16;11(12):e0168366. doi: 10.1371/journal.pone.0168366 (PMC5161367; doi:10.1371/journal.pone.0168366)
Supplement: S1 Fig — HTS were performed with B9 cells in duplicate in 384 wells plates. Wells from A1 to H2 were used as total control treated with vehicle 0.5% DMSO, while I1 to P2 were used as blank control (no B9 cells seeded in these wells). Puromycin controls were seeded in wells from A3 to P4, starting from 20 μM, 2 fold dilution down to 0.16 μM. Library compounds were seeded in wells from A5 to P24. (DOC) [file pone.0168366.s001.doc]

**Supporting information**

**S1 Fig. HTS plate configuration**

Compound 1 to compound 160 in duplicates

High throughput screening (HTS) was performed with B9 cells in duplicate in 384-well plates. Wells from A1 to H2 were used as total control treated with vehicle 0.5% DMSO, while wells I1 to P2 were used as blank controls (no B9 cells seeded in these wells). Puromycin controls were seeded in wells A3 to P4, starting from 20 µM with2-fold dilutions down to 0.16 µM. Library compounds were seeded in wells A5 to P24.
