# Supplementary material for: Discovery of Novel Small Molecule Inhibitors of VEGF Expression in Tumor Cells Using a Cell-Based High Throughput Screening Platform
Source: PLoS One. 2016 Dec 16;11(12):e0168366. doi: 10.1371/journal.pone.0168366 (PMC5161367; doi:10.1371/journal.pone.0168366)
Supplement: S2 Fig — Stable cell lines were generated for a high throughput screen to identify compounds that inhibit VEGF UTRs-mediated gene expression. Human embryonic kidney (HEK293) cells were transfected with the VEGF GEMS™ plasmid that contains a luciferase reporter driven by a CMV promoter and flanked with the VEGF 5’-UTR and the VEGF 3’-UTR. After two weeks of culture under the pressure of hygromycin (200 μg/mL) selection, nineteen resistant clones were expanded and screened for luciferase activity. The three clones with highest levels of luciferase activities were compared side by side. Luciferase activity was normalized against total protein concentration in the cell lysates. (DOC) [file pone.0168366.s002.doc]

**S2 Fig. Side-by-side comparison of VEGF GEMS^TM^ stable cell lines.**

Stable cell lines were generated for HTS to identify compounds that inhibit gene expression mediated by VEGF mRNA UTRs. Human embryonic kidney (HEK293) cells were transfected with the VEGF GEMS^TM^ plasmid that contains luciferase reporter driven by a human CMV (cytomegalovirus) promoter and flanked by the VEGF 5’- and 3’-UTRs. After culturing for two weeks under the pressure of hygromycin (200 µg/mL) selection, 19 resistant clones were expanded and screened for luciferase activity. The three clones with highest levels of luciferase activity were compared side by side. The results from the study indicated that cells of clone B9 had the highest level of luciferase activity when normalized to total protein concentration in the lysates.
