# Supplementary material for: Discovery of Novel Small Molecule Inhibitors of VEGF Expression in Tumor Cells Using a Cell-Based High Throughput Screening Platform
Source: PLoS One. 2016 Dec 16;11(12):e0168366. doi: 10.1371/journal.pone.0168366 (PMC5161367; doi:10.1371/journal.pone.0168366)
Supplement: S3 Fig — Puromycin inhibits reporter gene expression independent of the UTRs in the GEMS™ vector. The stable cell lines B9 and B12 used in this study were generated in HEK293 cells transfected with the constructs shown in the diagrams on the top of the graph. The activity of luciferase was measured with the substrate Bright-Glow (Promega). (DOC) [file pone.0168366.s003.doc]

**S3 Fig. Puromycin shows no selectivity in the VEGF-UTR selectivity assay.**

Puromycin inhibits reporter gene expression independent of the UTRs in the GEMS^TM^ vector. The stable cell lines B9 and B12 used in this study were generated in HEK293 cells transfected with the constructs shown in the diagrams on the top of the graph. The activity of luciferase was measured with the substrate Bright-Glow (Promega).
