# Supplementary material for: Discovery of Novel Small Molecule Inhibitors of VEGF Expression in Tumor Cells Using a Cell-Based High Throughput Screening Platform
Source: PLoS One. 2016 Dec 16;11(12):e0168366. doi: 10.1371/journal.pone.0168366 (PMC5161367; doi:10.1371/journal.pone.0168366)
Supplement: S4 Fig — PDE5 activity was measured with a PDE5 assay kit (cat#: R8039, Molecular Devices) according to manufacturer’s instructions. PDE5 enzyme was purchased from CalBiochem (cat#: 524715). Tadalafil (Cialis® was used as a positive control. Assays were performed in 96-well plates in duplicate, the fluorescence polarization was determined on a ViewLux microplate reader (Perkin Elmer) using the instrument settings recommended by the manufacturer. (DOC) [file pone.0168366.s004.doc]

**S4 Fig. PTC-510 does not inhibit the activity of phosphodiesterase 5 (PDE5).**

PDE5 activity was quantified usinga PDE5 assay kit (cat#: R8039, Molecular Devices) according to manufacturer’s instructions. PDE5 enzyme was purchased from CalBiochem (cat#: 524715). The positive control, tadalafil (Cialis®), was synthesized in house. Assays were performed in 96-well plates in duplicate and fluorescence polarization was determined on a ViewLux microplate reader (Perkin Elmer) using the instrument settings recommended by the manufacturer.

S5. Fig.

Selective inhibition by PTC-510 of reporter gene expression mediated by VEGF mRNA UTRs. The assays were performed in triplicate, and in each case the mean inhibition + SD (error bars) is shown in the figure. All luciferase reporter stable cell lines used in this study were generated from HEK 293 cells transfected with the luciferase reporter gene flanked with the UTRs derived from each target gene. HIF-1α: hypoxia Inducible Factor 1 alpha; DPPIV: dipeptidyl peptidase IV

**S6 Fig. Exposure of PTC-510 after oral administration**.

Male C57BL/6 mice were administered test compounds in 5% DMSO and 95% PEG300 at a dose of 10 mg/kg. At specified time points (3 mice per time point), mice were euthanized and blood collected by terminal cardiac puncture. Plasma concentrations of test compounds were then quantified by LC/MS-MS.

**S7 Fig. Body weight changes for mice in the xenograft study shown in Fig 4d.**

Body weight was measured at the indicated time for each mouse until the group average tumor size reached 1000 cm^3^ and the whole group were then took down. Data in this graph represents the average body weight (g) ± SD, n = 10.

**S1 Table**. Summary of PTC-510’s pharmacological properties

| **Test** | **Units** | **PTC-510** |
| --- | --- | --- |
| Molecular weight |  | 524 |
| cLogP |  | 5.35 |
| HeLa VEGF ELISA/SAR EC50 | nM | 6 |
| HeLa VEGF ELISA/SAR EC90 | nM | 50 |
| VEGF UTR specificity |  | yes |
| HeLa cytotoxicity 48 hr assay | CC50 nM | 200 |
| PBMC cytoxtoxicity | CC50 μM | >30 |
| Microsome metabolism - human | % loss @ 1 h | 0% |
| Microsome metabolism - primate | % loss @ 1 h | 0% |
| Microsome metabolism - dog | % loss @ 1 h | 0% |
| Microsome metabolism - rat | % loss @ 1 h | 0% |
| Microsome metabolism - murine | % loss @ 1 h | 0% |
| p450 inhibition Cyp3A4 | IC50 μM | **3.7** |
| p450 inhibition Cyp2C19 | IC50 μM | >5 |
| p450 inhibition Cyp1A2 | IC50 μM | >5 |
| Caco-2 | Papp cm/sec x10E-6 | **1.0** |
| HERG | % inhibition @ 10 μM | 2% |
| Acute Toxicity Dose (ATD) in mouse | mg/kg | No overt toxicity at 1000 mg/kg |
| Oral bioavailability in the rat in DMSO/PEG |  | 50% |
| Dose normalized AUC in the rat | µg.hr/L | 2.60 |

**Pharmacological characterization of PTC-510.** Pharmacological assays were performed to characterize PTC-510, including metabolic stability in human, primate mouse and rat liver microsomes; absorption in Caco-2 cells; *in vitro* effect on hERG ion channel; pharmacokinetics; and acute toxicity in the rat.

**S2 Table**

**Oral administration of PTC-510 selectively reduces levels of intratumor HT1080 tumor hVEGF in vivo**

**
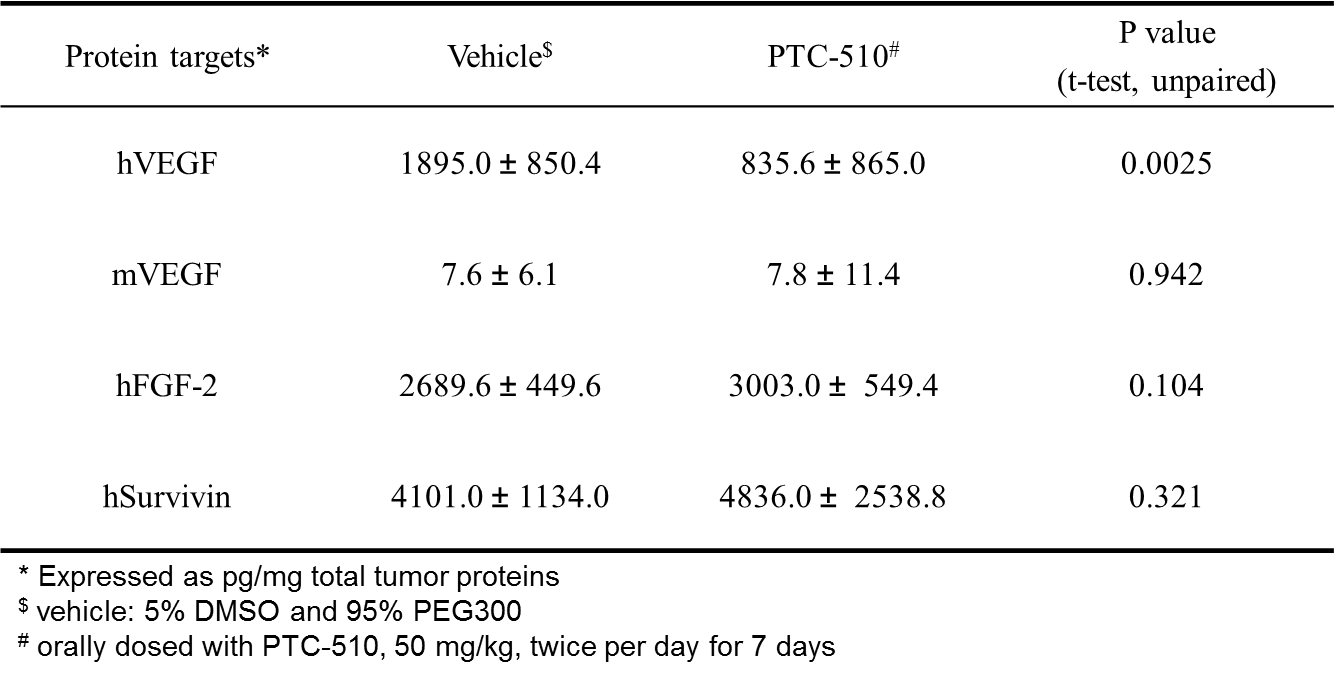
*** Expressed as pg/mg total tumor proteins (mean +/- SD, n = 10)

$ Vehicle: 5% DMSO and 95% PEG300

# Dosed with PTC-510, 50 mg/kg, twice per day for 7 days
