# Supplementary material for: Discovery of Novel Small Molecule Inhibitors of VEGF Expression in Tumor Cells Using a Cell-Based High Throughput Screening Platform
Source: PLoS One. 2016 Dec 16;11(12):e0168366. doi: 10.1371/journal.pone.0168366 (PMC5161367; doi:10.1371/journal.pone.0168366)
Supplement: S5 Fig — Selective inhibition by PTC-510 of reporter gene expression mediated by VEGF mRNA UTRs. The assays were performed in triplicate, and in each case the mean inhibition ± SD (error bars) is shown in the figure. All luciferase reporter stable cell lines used in this study were generated from HEK 293 cells transfected with the luciferase reporter gene flanked with the UTRs derived from each target gene. HIF-1α: hypoxia Inducible Factor 1 alpha; DPPIV: dipeptidyl peptidase IV. (DOC) [file pone.0168366.s005.doc]

**S5 Fig. PTC-510 preferentially inhibited VEGF-UTRs-driven reporter gene expression**

Selective inhibition by PTC-510 of reporter gene expression mediated by VEGF mRNA UTRs. The assays were performed in triplicate, and in each case the mean inhibition + SD (error bars) is shown in the figure. All luciferase reporter stable cell lines used in this study were generated from HEK 293 cells transfected with the luciferase reporter gene flanked with the UTRs derived from each target gene. HIF-1α: hypoxia Inducible Factor 1 alpha; DPPIV: dipeptidyl peptidase IV.
