# Supplementary material for: Discovery of Novel Small Molecule Inhibitors of VEGF Expression in Tumor Cells Using a Cell-Based High Throughput Screening Platform
Source: PLoS One. 2016 Dec 16;11(12):e0168366. doi: 10.1371/journal.pone.0168366 (PMC5161367; doi:10.1371/journal.pone.0168366)
Supplement: S6 Fig — Male C57BL/6 mice were dosed with test compound in 5% DMSO and 95% PEG300. At specified time points (3 mice per time point), mice were euthanized and blood collected by terminal cardiac puncture. Plasma test compounds were then measured by LC/MS-MS. (DOC) [file pone.0168366.s006.doc]

**S6 Fig. Exposure of PTC-510 after oral administration**.

Male C57BL/6 mice were dosed with test compound in 5% DMSO and 95% PEG300. At specified time points (3 mice per time point), mice were euthanized and blood collected by terminal cardiac puncture. Plasma test compounds were then measured by LC/MS-MS.
