# Supplementary material for: Discovery of Novel Small Molecule Inhibitors of VEGF Expression in Tumor Cells Using a Cell-Based High Throughput Screening Platform
Source: PLoS One. 2016 Dec 16;11(12):e0168366. doi: 10.1371/journal.pone.0168366 (PMC5161367; doi:10.1371/journal.pone.0168366)
Supplement: S7 Fig — Body weight was measured at the indicated time for each mouse until the group average tumor size reached 1000 cm3 and the whole group were then took down. (DOC) [file pone.0168366.s007.doc]

**S7 Fig. Body weight changes for mice in the xenograft study shown in Fig 4d.**

Body weight was measured at the indicated time for each mouse until the group average tumor size reached 1000 cm^3^ and the whole group were then took down. Data in this graph represents the average body weight (g) ± SD, n = 10.
