# Supplementary material for: Discovery of Novel Small Molecule Inhibitors of VEGF Expression in Tumor Cells Using a Cell-Based High Throughput Screening Platform
Source: PLoS One. 2016 Dec 16;11(12):e0168366. doi: 10.1371/journal.pone.0168366 (PMC5161367; doi:10.1371/journal.pone.0168366)
Supplement: S1 Table — (DOC) [file pone.0168366.s008.doc]

**S1 Table. Summary of PTC-510’s pharmacological properties**

| **Test** | **Units** | **PTC-510** |
| --- | --- | --- |
| Molecular weight |  | 524 |
| cLogP |  | 5.35 |
| HeLa VEGF ELISA/SAR EC50 | nM | 6 |
| HeLa VEGF ELISA/SAR EC90 | nM | 50 |
| VEGF UTR specificity |  | yes |
| HeLa cytotoxicity 48 hr assay | CC50 nM | 200 |
| PBMC cytoxtoxicity | CC50 μM | >30 |
| Microsome metabolism - human | % loss @ 1 h | 0% |
| Microsome metabolism - primate | % loss @ 1 h | 0% |
| Microsome metabolism - dog | % loss @ 1 h | 0% |
| Microsome metabolism - rat | % loss @ 1 h | 0% |
| Microsome metabolism - murine | % loss @ 1 h | 0% |
| p450 inhibition Cyp3A4 | IC50 μM | **3.7** |
| p450 inhibition Cyp2C19 | IC50 μM | >5 |
| p450 inhibition Cyp1A2 | IC50 μM | >5 |
| Caco-2 | Papp cm/sec x10E-6 | **1.0** |
| HERG | % inhibition @ 10 μM | 2% |
| Acute Toxicity Dose (ATD) in mouse | mg/kg | No overt toxicity at 1000 mg/kg |
| Oral bioavailability in the rat in DMSO/PEG |  | 50% |
| Dose normalized AUC in the rat | µg.hr/L | 2.60 |

Pharmacological assays were performed to characterize PTC-510, including metabolic stability in human, primate mouse and rat liver microsomes; absorption in Caco-2 cells; *in vitro* effect on hERG ion channel; pharmacokinetics; and acute toxicity in the rat.
