# Supplementary material for: Discovery of Novel Small Molecule Inhibitors of VEGF Expression in Tumor Cells Using a Cell-Based High Throughput Screening Platform
Source: PLoS One. 2016 Dec 16;11(12):e0168366. doi: 10.1371/journal.pone.0168366 (PMC5161367; doi:10.1371/journal.pone.0168366)
Supplement: S2 Table — (DOC) [file pone.0168366.s009.doc]

**S2 Table. Oral administration of PTC-510 selectively reduces levels of intratumor HT1080 tumor hVEGF in vivo**

**
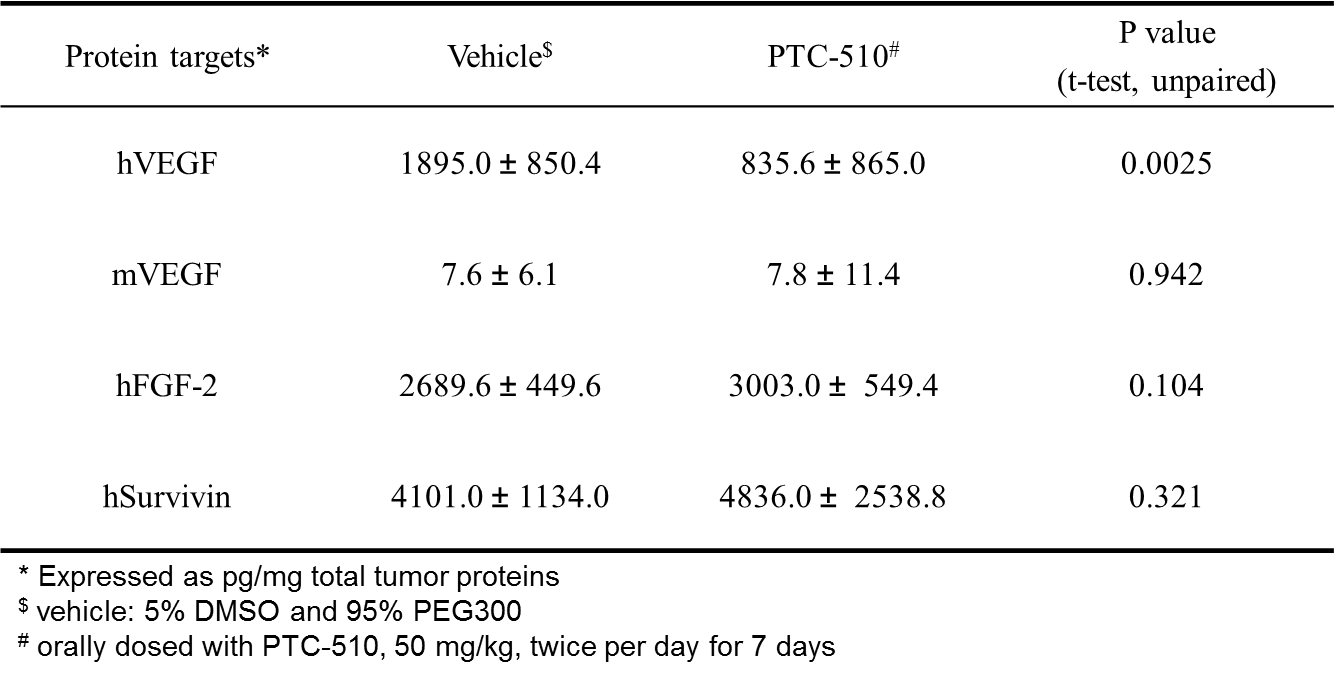
*** Expressed as pg/mg total tumor proteins (mean +/- SD, n = 10)

$ Vehicle: 5% DMSO and 95% PEG300

# Dosed with PTC-510, 50 mg/kg, twice per day for 7 days
